# Evaluation of the Association of Omentin 1 rs2274907 A>T and rs2274908 G>A Gene Polymorphisms with Coronary Artery Disease in Indian Population: A Case Control Study

**DOI:** 10.3390/jpm9020030

**Published:** 2019-06-06

**Authors:** Chandan K Jha, Rashid Mir, Imadeldin Elfaki, Jamsheed Javid, Abdullatif Taha Babakr, Shaheena Banu, S. M. S. Chahal

**Affiliations:** 1 Department of Human Genetics, Punjabi University, Punjab 147002, India; chandujha58@gmail.com; 2 Prince Fahd Bin Sultan Research chair, Department of Medical Lab Technology, Faculty of Applied Medical Sciences, University of Tabuk, Tabuk 71491, Saudi Arabia; rashid@ut.edu.sa (R.M.); jamsheedjavidsjj@gmail.com (J.J.); 3 Department of Biochemistry, Faculty of Science, University of Tabuk, Tabuk 71491, Saudi Arabia; ielfaki@ut.edu.sa; 4 Department of Medical Biochemistry, Faculty of Medicine, Umm Al-Qura University, Makkah 57039, Saudi Arabia; abdullatiftaha@yahoo.com; 5 Sri Jayadeva Institute of Cardiovascular science and Research, Bangalore 560069, India; shanbanubanu@gmail.com

**Keywords:** omentin, intelectin, rs2274907 and rs2274908 SNPs, allele specific PCR, ARMS-PCR, Coronary Artery Disease

## Abstract

Coronary artery disease (CAD) is a major cause of death all over the world. CAD is caused by atherosclerosis which is induced by the interaction of genetic factors and environmental factors. Traditional environmental risk factors include hyperlipidemia, diabetes mellitus, lack of exercise, obesity, poor diet and others. Genome-wide association studies have revealed the association of certain gene polymorphisms with susceptibility to CAD. Omentin 1 is an adipokine secreted by the visceral adipose tissues and has been reported to have anti-inflammatory, cardioprotective, and enhances insulin sensitivity. In this study, we examined the role of omentin-1 common single nucleotide polymorphisms (SNPs) (rs2274907 A>T and rs2274908 G>A) in CAD. We genotyped 100 CAD patients and 100 matched healthy controls from the south Indian population using an amplification refractory mutation system (ARMS-PCR) and allele-specific PCR (AS-PCR). Our result indicated the rs2274908 G>A is not associated with CAD. Results showed that there was a significant difference in rs2274907 A>T genotype distribution between controls and CAD cases (*P*-value < 0.05). Results indicated that the AT genotype of the rs2274907 is associated with CAD with OR = 3.0 (95% confidence interval (CI), 1.64 to 5.49), 1.65 (1.27 to 2.163), *P* = 0.002. The T allele of the rs2274907 was also associated with CAD with OR = 1.82 (95% CI, 1.193 to 2.80), 1.37 (1.08 to 1.74), *P* = 0.005. Rs2274907 genotype distribution was also correlated with serum total cholesterol, high-density lipoprotein cholesterol (HDL-C), low-density lipoprotein cholesterol (LDL-C), hypertension and diabetes. We conclude that the AT genotype and the T allele of the rs2274907 A>T is associated with Cad in the south Indian population. Further studies on the effect of the rs2274907 A>T on omentin-1 function are recommended, and future well-designed studies with larger sample sizes and in different populations are required to validate our findings.

## 1. Introduction 

**Figure 1:**
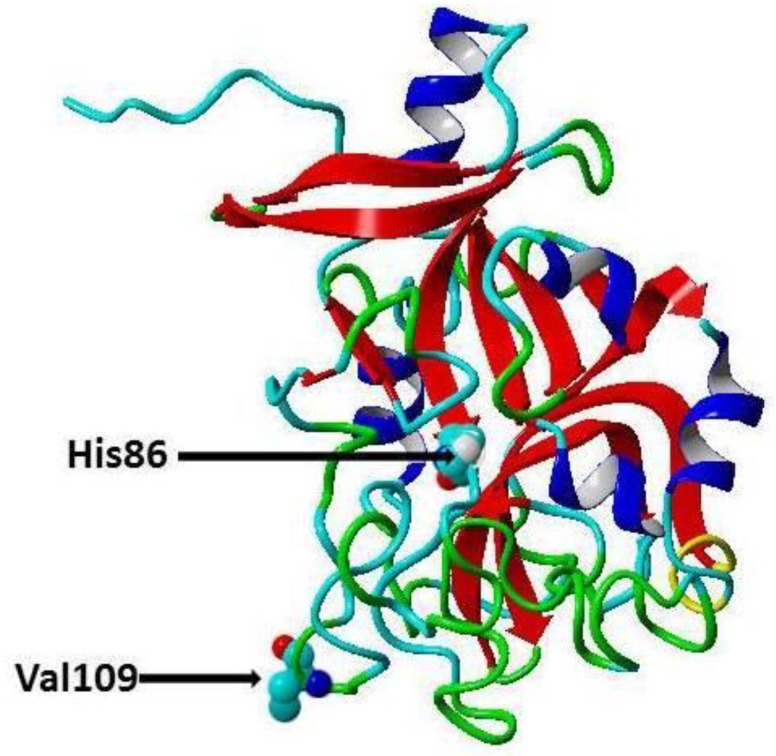
Secondary cartoon structure of human omentin (PDB ID 4WMQ [19]. The amino acid residues, Valine 109 (rs2274907) and the Histidine 86 (rs2274908), are shown in atom presentation. This figure has been prepared using YASARA View (version 17.7.30).

Cardiovascular diseases (CVDs) are one of the major causes of hospitalization and death and account for about 31% of deaths worldwide [1]. In India, about 25% of the deaths are caused by CVDs, and about 50% of CVD deaths occur prior to the age of 70 years, while in Europe about 25% of CVD deaths occur before this age [2], thus, CVDs affect the Indian population in the most productive years of their lives [2]. CVDs are a group of pathological conditions that include atherosclerosis, coronary artery disease (CAD), angina, myocardial infarction, rheumatic and congenital heart diseases, stroke and venous thromboembolism [3]. Traditional risk factors for CVD include age, low high-density lipoprotein cholesterol (HDL-C), high low-density lipoprotein cholesterol (LDL-C), hypercholesterolemia, Type 2 diabetes (T2D), poor diet, elevated blood pressure, obesity, physical inactivity, and smoking [4]. Recently, it has been proposed there are genetic risk factors for CVD, and these risk factors can be identified through robust genome-wide association studies (GWASs) [5]. Moreover, GWASs revealed that there are certain single nucleotide polymorphisms (SNPs) significantly associated with specific disorders in certain populations [6,7,8]. For instance, transforming growth factor-β1 and microRNA gene variations have been shown to be associated with CAD in Chinese, Caucasian [9], and Indian cohorts, respectively [10,11,12,13]. Omentin protein (also called intelectin) is a novel adipocytokine secreted by visceral adipose tissues (e.g., retroperitoneal and epicardial fat), intestinal Paneth cells and endothelial cells [14]. It is composed of 313 amino acid residues (35 kDa) and circulates in the human bloodstream. There are two isoforms of omentin with amino acid sequence similarity about 83% [14]. Omentin1 is the major isoform in blood and studied more than omentin 2 [14]. Omentin1 has been shown to reduce the inflammatory response, and it has been found that CAD patient’s exhibit reduced levels of omentin1 in the circulation and in epicardial adipose tissue [15]. Furthermore, it has been reported that blood omentin1 inversely correlates with obesity, fasting insulin and leptin levels, while positively correlates with HDL-C and adiponectin [14]. Moreover, it was demonstrated that omentin enhances insulin sensitivity and that omentin protects against obesity and its comorbidities such as Type 2 diabetes (T2D) and CVDs [16]. Recently, Omentin-1 Val109Asp gene polymorphism has been shown to be associated with CAD in Pakistani and Iranian populations [17,18]. In the present study, we have examined the association of common polymorphisms [19] in the omentin gene, the rs2274907 A>T (Val109Asp) and rs2274908 G>A (His86His) (Figure 1) with CAD in the Indian population.

## 2. Materials and Methods

### 2.1. Criteria for Selection of Patients and Controls 

We selected patients undergoing elective angiography for the examination of stable chest pain at the Institute of Cardiovascular Science and Research, Karnataka. Some non-invasive tests were conducted, for example, an electrocardiogram (ECG), Holter monitoring, chest X-ray, echocardiogram (echo), cardiac computed tomography (CCT), exercise stress test and myocardial perfusion imaging (MPI) or equilibrium radionuclide angiogram.

### 2.2. Inclusion Criteria for Patient Selection 

Patients with new-onset acute chest pain undergoing coronary angiography were selected in this study. Subjects were classified based on their coronary angiographic findings as either significant CAD (stenosis ≥ 50%) that were included, or Intracranial atherosclerotic disease (ICAD) (no stenosis or stenosis < 50%) that were not included. 

### 2.3. Exclusion Criteria

Patients with a history of non-coronary cardiac disorders, percutaneous transluminal coronary angioplasty (PTCA), or previously performed coronary bypass surgery have been excluded due to their treated coronary status. 

### 2.4. Selection of Healthy Controls 

They were selected from visitors at the Institute of Cardiovascular for a routine checkup. The healthy controls selected were with no previous heart attack or angina. Some of the blood biochemistry analyses were also performed on healthy controls. All subjects completed the questionnaire as well as informed consent. This study has been approved by the Institutional Ethics Committee of Punjabi University.

### 2.5. Collection of Case Histroy and Measurement of Certein Biochemical Parametes that are Related to CAD

After overnight fasting and prior to coronary angiography, patient case history was collected in addition to a blood sample from each subject for measurement of random blood glucose, total cholesterol, Triacylglycerol, high-density lipoprotein-cholesterol (HDL-C), and low-density lipoprotein cholesterol (LDL-C) concentrations and total cholesterol/HDL-C ratios have been assayed using the standard protocols.

### 2.6. Sample Collection and DNA Extraction

From each Subject about 4 mL, a peripheral blood sample was collected in an EDTA tube. Genomic DNA was isolated using the Thermo Scientific Genomic DNA Purification Kit (USA) from the whole blood according to the manufacturer’s instructions. The DNA integrity was checked with 0.8 agarose gel electrophoresis and Nanodrop. 

### 2.7. Detection of ITLN1 rs2274907 A>T rs2274908 G>A Gene Polymorphisms by ARMS-PCR and (AS) PCR 

**Figure 2:**
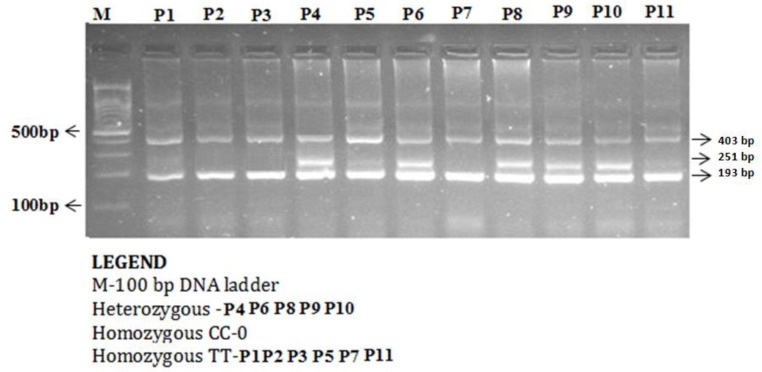
Detection of Omentin-1 rs2274907 A>T gene variation with amplification refractory mutation system (ARMS-PCR) in coronary artery disease (CAD) cases.

**Figure 3:**
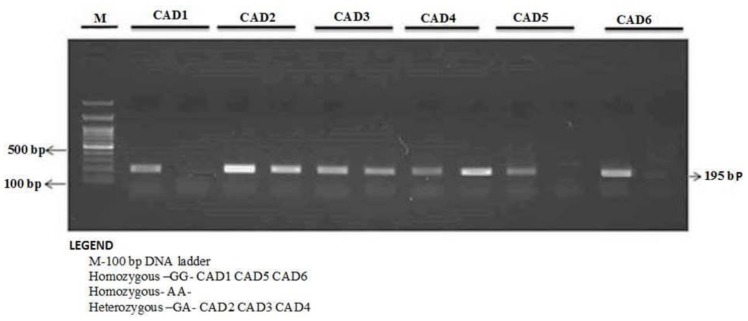
Detection of Omentin-1 rs2274908 G>A gene variation with allele-specific PCR (AS-PCR) in CAD cases.

The enzyme-coding region of ITLN1 (Omentin) gene contains well-investigated single nucleotide polymorphisms (rs2274907 and rs2274908). The ARMS- primers were designed by Primer3 Software. For the rs2274907, the primers mixture FO/R0 mixture generates a band of 403 bp as a control band, FO/RI produce a band of 193 bp for A allele, whereas, FI/RO mixture generates a band of 251 bp for T allele (Table 1, Figure 2). The primers sequences were shown in Table 1. For the rs2274908 G>A, the primers ITLN1-F1/ ITLN1-R mixture generates a wild band of 195 bp. The primers ITLN1-F2/ ITLN1-R mixture generates a mutant band of 195bp (Table 1, Figure 3). 

The ARMS-PCR reaction was performed in a 25-μL reaction mixture containing 100 ng template DNA, 0.25 μL of 25 pmol of each primer, 2.5 μL 10 mM dNTP’s 1.5 μL of 20 mM MgCl_2_, and 0.3 μL of 5 U/μL Taq polymerase with 2.5 μL of 10× Taq Buffer (Fermantas, Waltham, MA, USA). The thermocycling conditions were initial denaturation at 96 °C for 10 min and 30 cycles: 96 °C for 30 s, 60 °C for 45 s, 72 °C for 45 s, and final extension at 72 °C for 5 min. The PCR products were analyzed using 1.5% agarose gel electrophoresis. After electrophoresis, amplified PCR products were visualized using ultraviolet trans-illuminator as depicted in Figure 2 and Figure 3. 

### 2.8. Statistical Analysis

The statistical analyses were performed with the SPSS 16.0 software package. We used the Chi-square analysis and Fisher exact test to compare Omentin 1 rs2274907 and rs2274908 Gene polymorphisms frequency with several clinical aspects, for examples the age, gender, serum cholesterol, HDL-C, LDL-C, triglyceride (TG), hypertension, diabetes, alcohol and smoking. The associations between rs2274907 genotypes and risk of CAD patients were estimated by calculating odds ratios (ORs), risk ratios (RRs) and risk differences (RDs) with 95% confidence intervals (CIs). The *P*-value < 0.05 was considered to be significant.

## 3. Results

### 3.1. Baseline Characteristics of CAD Patients and Controls

Clinical characteristics of patients are shown in Table 2. The study included 100 CAD patients (88 males and 12 females), and 100 matched healthy control controls. Among the 100 CAD patients, there were 47 patients were ≤50 years old, while 53 patients >50 years old as depicted in (Table 2).

### 3.2. The rs2274907 A>T Genotype Distribution in CAD Cases and Controls

In CAD patients, the AA, AT and TT genotype frequencies were 24, 75 and 1%, respectively whereas in healthy controls the AA, AT and TT genotype frequencies were 49%, 51%, and 0%, respectively (Table 3). There was significant genotype distribution between the cases and the healthy controls (*P* = 0.009, Table 3). Among patients there was high percentage of heterozygosity 75% compared to 51% in controls. 

### 3.3. Some Clinical Characteristics of CAD Patients 

Results also indicated that in the CAD patients there were 64, 81, 67, 72, and 58% had random blood sugar (RBS), total Cholesterol, HDL-C, LDL-C, TGL below or equal to 140 mg, 200 mg, 40 mg, 100 mg, 150 mg respectively. Whereas, 36, 19, 33, 28, 42% of CAD cases had greater than 140 mg, 200 mg, 40 mg, 100 mg, 150 mg of RBS, total cholesterol, HDL-C, LDL-C, TGL respectively (Table 4). Results showed that in CAD cases there were 22, and 16% were positive, while in 78, 84% were negative for hypertension and T2D, respectively. In addition, 57% and 35% of CAD cases were smokers and alcoholic, respectively, and 43% and 65% of subjects were non-smokers and nonalcoholic, respectively (Table 4).

### 3.4. Association with Gender and Age

Results showed that the rs2274907 genotype distribution and gender and age were not significantly associated with *P*-value > 0.05, Table 4). 

### 3.5. Correlations with Random Blood Sugar (RBS), Plasma Cholesterol

**Figure 4:**
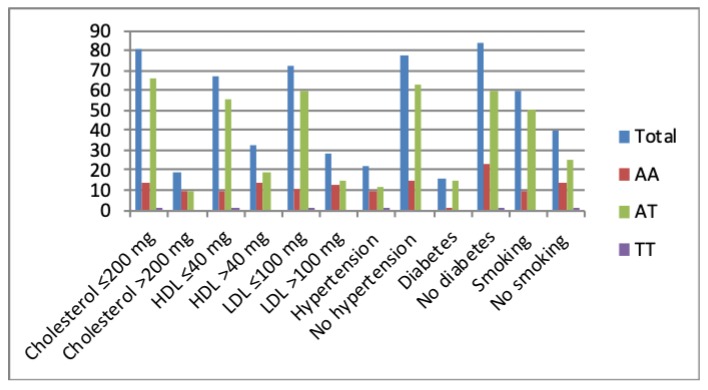
The rs2274907 A>T genotype distribution with an elevated or normal lipid profile, presence or absence of hypertension and diabetes, smoking or no smoking.

Results indicated that there is no association between RBS and rs2274907 genotype distribution (*P*-value > 0.05, Table 4). Results also showed that patients with the AT genotype were significantly more susceptible to CAD than the AA or TT genotypes in normal or elevated lipid profiles, diabetes, no diabetes, hypertension, no hypertension, smoking or no smoking (Figure 4, Table 4).

### 3.6. Correlation with Hypertension and Diabetes

Results indicated that hypertension and diabetes were significantly associated with rs2274907 genotype distribution (*P*-values < 0.05, Table 4).

### 3.7. Correlation with Smoking and Alcohol Intake

Our results indicated that rs2274907 genotype distribution is significantly associated with smoking (*P*-value < 0.05), but the not with alcohol intake (*P*-value > 0.05, Table 4). 

### 3.8. Association of the rs2274907 A>T Gene Polymorphism with CAD 

Our results indicated that the AT genotype of the rs2274907 is associated with CAD with OR = 3.0 (95% CI, 1.64 to 5.49), 1.65 (1.27 to 2.163), *P* = 0.002 (Table 5). The T allele of the rs2274907 was also associated with CAD with OR = 1.82 (95% CI, 1.193 to 2.80), 1.37 (1.08 to 1.74), *P* = 0.005 (Table 5). 

### 3.9. The rs2274908 G>A Genotype Distribution in CAD Cases and Controls

Our results showed that there was no significant difference in the rs2274908 G>A genotype distribution between patients and healthy controls, *P*-value >0.05 (Table 6).

### 3.10. Association of rs2274908 G>A Polymorphism with the Susceptibility to CAD Patients

Our result indicated that the GA genotype of the rs2274908 is not associated with CAD with OR = 0.70 (95% CI, 0.344 to 1.45), RR = 0.83(0.56 to 1.24), *P*-value 0.34 (Table 7). The A allele of the rs2274908 was also not associated with CAD with OR = 0.92 (95% CI, 0.61 to 1.37), 0.95 (0.78 to 1.16), *P* = 0.68 (Table 7).

## 4. Discussion

CVDs are an important cause of death and morbidity caused mainly by atherosclerosis [20]. Atherosclerosis is a local inflammation in the arterial wall characterized by lipid retention, smooth muscle cell proliferation, apoptosis, and fibrous cap formation [21]. It is a chronic inflammatory disease initiated by the infiltration and retention of cholesterol in the form of LDL-C in the arterial wall [21,22]. LDL-C retention leads to injury of arterial endothelial cells and infiltration of monocytes and macrophages. Then macrophage phagocytes lipoprotein and forms the foam cells, which is a characteristic of the fatty streak phase of atherosclerosis [21,22]. Then the macrophage promotes the oxidative stress and secretion of chemokine and cytokines which result in enhanced inflammatory response leading to the formation of the atherosclerotic plaque [21]. Eventually, rupture of the fibrous cap of the plaque results in thrombosis and ischemic heart disease [22]. Du et al, 2016 reported that in CAD patients, there were lowered omentin-1 levels in plasma and in epicardial adipose tissue compared to controls without CAD [15]. Moreover, it was suggested that reduced plasma omentin levels can be used as a biomarker for early atherosclerosis and metabolic risk factors [23,24]. In contrast, Saely et al, 2016, have reported that plasma levels of omentin are increased in patients with CAD [25]. It has been reported that Omentin protects against inflammation, atherosclerosis and T2D (16). The rs2274907A>T is located in the fourth exon of ITLN1 gene and results in substitution Valine 109 to Aspartic acid (Val109Asp) (Figure 1) [26]. The second polymorphism (rs2274908 G>A) is located also in the fourth exon and results in no change of the amino acid residue histidine 86 (His86His) (Figure 1) [26]. Valine is a hydrophobic amino acid, while aspartic acid is a negatively charged amino acid, therefore, the rs2274907 may influence the omentin function. However, this must await future functional studies for omentin protein. Our results indicated that the AT genotype of the rs2274907A>T is associated with increased susceptibility to CAD with OR = 3.0 (95% CI, 1.64 to 5.49), RR = 1.65(1.27 to 2.163), *P* = 0.002 (Table 5). Likewise, the T allele of the rs2274907A>T is associated with increased risk to CAD with OR = 1.82 (95%, 1.193 to 2.80), RR =1.37 (1.08 to 1.74), *P*-value = 0.005 (Table 5). Our results also showed that there were significant differences between the rs2274907A>T SNP genotype distribution and serum levels of total cholesterol, LDL-C, HDL-C, presence or absence of hypertension or diabetes, and smoking or no smoking (Table 4). The results also suggested that cases that were carriers of AT genotype of the rs2274907A>T were significantly more susceptible to CAD in cases of hyperlipidemia, normal lipid profile, presence or absence of diseases, smoking, or no smoking (Table 4, Figure 4). These results may be in agreement with the recent study by Jamshidi et al., 2017, who reported that the T allele of the rs2274907 A>T is associated with increased risk to CAD in males of the Iranian population [17], nevertheless, we could not see a significant difference between males and females in the south Indian cohort (*P*-value > 0.05, Table 4). This discrepancy is probably due to different sample size and ethnicity. Our result also showed that the AT genotype of the rs2274907 is significantly more frequent in CAD patients than in controls (*P*-value < 0.05, Table 3). This result is also in agreement with a recent study conducted in Pakistani population and demonstrated that AT genotype (Val/Asp (heterozygous mutant) of the rs2274907 is an association with the risk of CAD [18]. Our result also may be in accordance with previous findings suggesting that omentin has a cardioprotective effect [27] as a nitric oxide-mediated vasodilator [28], and that the plasma omentin is negatively associated with carotid intima-media thickness [23]. Our results indicated that there a significance difference in the genotype distribution of rs2274907A>T between cases with diabetes and cases with no diabetes (Table 4). This result suggested that the rs2274907A>T may play a role in the induction of diabetes. This result may be consistent with a previous study that suggested serum omentin1 levels are negatively correlated with insulin resistance and T2D [29]. Our results also indicated that the rs2274908 G>A is not associated with CAD (Table 6 and Table 7). This result is expected since the SNP (rs2274908 G>A) results show no change of the amino acid (His86His), (Figure 1), and therefore, it has no influence on omentin1 structure and function. Atherosclerosis (and its clinical manifestations such as CAD and stroke) is a multifactorial disease induced from the interaction of environmental and genetic factors [5], our result would help in identification and stratification of the susceptible groups to atherosclerosis, as reduction of the risk factors by modification of lifestyle, lipid-lowering treatment, control of blood glucose and pressure are effective ways for preventing or delaying atherosclerosis [30]. 

## 5. Conclusions

The result of the present study suggests that the AT genotype and the T allele of the SNP rs2274907A>T are associated with increased risk of CAD. Future studies on the effect of rs2274907A>T on the function of omentin-1 are still required. Furthermore, well-designed studies with larger sample sizes and in different populations are recommended to validate these results.

## Figures and Tables

**Table 1:** Primers sequences of ARMS-PCR and Allele Specific-PCR.

Primers sequences of ARMS-PCR for Genotyping of rs2274907 A>T				
Fo-outer primer	F1	5-ACCCCTACCTTCCAGCCATCCC-3	403 bp	60 °C
Ro-outer primer	R1	5-CATGGGGCTGAAATGAACCCTCAGC-3		
FI-T-inner primer	T allele	5-TGCCGTCCCCCTCTGGGTAGT-3	251 bp	
RIA-inner primer	A allele	5-GTCAGCAGGGCAGCAAAGCAGA-3	193 bp	
**Primers sequence of Allele Specific -PCR for genotyping of rs2274908 G>A**				
ITLN1-F1 Wild	G allele	ACTTCCCACGCATGTCATTCTCG	195 bp	63 °C
ITLN1-F2 mutant	A allele	ACTTCCCACGCATGTCATTCTCA	195 bp	
ITLN1-R common	Reverse	CTTTCTTGTCATGGGGCTGAAATGAAC		

**Table 2:** Baseline characteristics of coronary artery disease (CAD) patients and controls.

Variables	No. of CAD Cases (*N* = %)		No. of Healthy Controls (*N* = %)
**No-of cases**	100 (100%)	**No-of controls**	100 (100%)
**Males**	88 (88%)	**Males**	85 (85%)
**Females**	12(12%)	**Females**	15 (15%)
**Age ≤ 50**	47(47%)	**Age ≤ 50**	45 (45%)
**Age > 50**	53(53%)	**Age > 50**	55 (55%)

**Table 3:** The rs2274907 A>T genotype distribution in CAD cases and controls.

Allele/Genotype	*N*	AA	%	A/T	%	TT	%	Chi-square	Degree of Freedom (DF)	*P*-Value
CAD patients	100	24	24%	75	75%	1	1%	14.13	2	0.009
Healthy controls	100	49	49%	51	51%	0	0%			

**Table 4:** Association of rs2274907 A>T genotype distribution with CAD clinical characteristics.

Subjects	*N* = 100	AA	AT	TT	X2	DF	*P*-Value
Association with gender							
Males	88	19	68	1	2.41	2	0.29
Females	12	5	7	0			
Association with Age							
Age ≤ 50	47	14	32	1	2.93	2	0.23
Age > 50	53	10	43	0			
Association with RBS							
RBS ≤ 140 mg	64	14	50	0	1.1	2	0.371
RBS > 140 mg	36	10	25	1			
Association with Cholesterol							
Cholesterol ≤ 200 mg	81	14	66	1	10.63	2	0.0049
Cholesterol > 200 mg	19	10	9	0			
Association with HDL							
HDL ≤ 40 mg	67	10	56	1	9.45	2	0.008
HDL > 40 mg	33	14	19	0			
Association with LDL							
LDL ≤ 100 mg	72	11	60	1	10.92	2	0.004
LDL > 100 mg	28	13	15	0			
Association with TGL							
TGL ≤ 150 mg	58	14	44	0	1.42	2	0.49
TGL > 150 mg	42	10	31	1			
Association with hypertension							
Hypertension	22	9	12	1	8.48	2	0.014
No hypertension	78	15	63	0			
Association with Diabetes							
Diabetes	16	1	15	0	8.16	2	0.016
No Diabetes	84	23	60	1			
Association with family history of CHD							
Coronary heart disease (CHD)	10	6	4	0	1.4	2	0.56
No CHD	90	18	71	1			
Association with Smoking							
Smoking	60	10	50	0	6.25	2	0.041
No Smoking	40	14	25	1			
Association with Alcohol							
Alcohol	35	10	25	0	1.1	2	0.57
No Alcohol	65	14	50	1			
Association with Pan Masala							
Pan Masala	2	1	1	0	0.77	2	0.68
No Pan Masala	98	23	74	1			

**Table 5:** Association of omentin rs2274907A>T gene variation with CAD.

Genotypes	Healthy Controls	CAD Cases	OR (95% CI)	Risk Ratio (RR)	*P*-Value
	(*N* = 100)	(*N* = 100)			
**Codominant**					
ITLN1-AA	49	24	1 (ref.)	1 (ref.)	
ITLN1-AT	51	75	3.0 (1.64 to 5.49)	1.65 (1.27 to 2.163)	0.002
ITLN1-TT	0	1	6.0 (0.23 to 154.31)	2.67 (0.24 to 29.66)	0.422
**Dominant**					
ITLN1-AA	49	24	1 (ref.)	1 (ref.)	
ITLN1-(AT + TT)	51	76	3.0 (1.66 to 5.56)	1.67 (1.28 to 2.18)	0.003
Recessive					
ITLN1-(AA + AT)	100	99	1 (ref.)	1 (ref.)	
ITLN1-TT	0	1	3.03 (0.12 to 75.28)	2.0 (0.181 to 22.255)	0.49
Allele					
ITLN1-A	149	123	1 (ref.)	1 (ref.)	
ITLN1-T	51	77	1.82 (1.193 to 2.80)	1.37 (1.08 to 1.74)	0.005

**Table 6:** The rs2274908 G>A genotype distribution in CAD cases and controls.

Subjects	*N*	GG	%	G/A	%	AA	%	Chi-Square	DF	*P*-Value
CAD patients	100	21	21%	78	78%	1	1%	1.9	2	0.386
Healthy controls	100	16	16%	84	84%	0	0%			

**Table 7:** Association of omentin rs2274908 G>A gene variation with the susceptibility to CAD patients.

Genotypes	Healthy Controls	CAD Cases	OR (95% CI)	Risk Ratio (RR)	*P*-Value
	(*N* = 100)	(*N* = 100)			
**Codominant**					
ITLN1-GG	16	21	1 (ref.)	1 (ref.)	
ITLN1-GA	84	78	0.70 (0.344 to 1.45)	0.83 (0.56 to 1.24)	0.34
ITLN1-AA	0	1	2.30 (0.08 to 60.23)	1.73 (0.15 to 19.68)	0.61
**Dominant**					
ITLN1-GG	16	21	1 (ref.)	1 (ref.)	
ITLN1-(GA + AA)	84	79	0.71 (0.34 to 1.47)	0.83 (0.56 to 1.24)	0.36
Recessive					
ITLN1-(GG + GA)	100	99	1 (ref.)	1 (ref.)	
ITLN1-AA	0	1	3.03 (0.12 to 75.28)	2.0 (0.181 to 22.255)	0.49
Allele					
ITLN1-G	116	120	1 (ref.)	1 (ref.)	
ITLN1-A	84	80	0.92 (0.61 to 1.37)	0.95 (0.78 to 1.16)	0.68

## References

[1] Stewart J., Manmathan G., Wilkinson P. (2017). Primary prevention of cardiovascular disease: A review of contemporary guidance and literature. JRSM Cardiovasc. Dis..

[2] Prabhakaran D., Jeemon P., Roy A. (2016). Cardiovascular diseases in India: Current epidemiology and future directions. Circulation.

[3] Mendis S., Puska P., Norrving B., World Health Organization (2011). Global Atlas on Cardiovascular Disease Prevention and Control Policies, Strategies and Interventions.

[4] Liao K.P., Solomon D.H. (2013). Traditional cardiovascular risk factors, inflammation and cardiovascular risk in rheumatoid arthritis. Rheumatology.

[5] Knowles J.W., Ashley E.A. (2018). Cardiovascular disease: The rise of the genetic risk score. PLoS Med..

[6] Price A.L., Spencer C.C., Donnelly P. (2015). Progress and promise in understanding the genetic basis of common diseases. Proc. Biol. Sci..

[7] Elfaki I., Almutairi F.M., Mir R., Khan R., Abu-Duhier F. (2018). Cytochrome P450 CYP1B1*2 gene and its association with T2D in Tabuk population, northwestern region of Saudi Arabia. Asian J. Pharm. Clin. Res..

[8] Elfaki I., Mir R., Almutairi F.M., Duhier F.M.A. (2018). Cytochrome P450: Polymorphisms and roles in cancer, diabetes and atherosclerosis. Asian Pac. J. Cancer Prev..

[9] Li Y.Y., Zhou Y.H., Gong G., Geng H.Y., Yang X.X. (2017). TGF-beta1 Gene -509C/T polymorphism and coronary artery disease: An updated meta-analysis involving 11,701 subjects. Front Physiol..

[10] Mir R., Jha C.K., Elfaki I., Rehman S., Javid J., Khullar N., Banu S., Chahal S. (2018). MicroRNA-224 (rs188519172 A>G) gene variability is associated with a decreased susceptibility to coronary artery disease: A case-control study. MicroRNA.

[11] Jha C.K., Mir R., Elfaki I., Khullar N., Rehman S., Javid J., Banu S., Chahal S. (2018). Potential impact of microRNA-423 gene variability in coronary artery disease. Endoc. Metab. Immune Disord. Drug Targ..

[12] Jha C.K., Mir R., Khullar N., Banu S., Chahal S.M.S. (2018). LDLR rs688 TT genotype and T allele are associated with increased susceptibility to coronary artery disease—A case-control study. J. Cardiovasc. Dev. Dis..

[13] Mir R., Jha C.K., Elfaki I., Javid J., Rehman S., Khullar N., Banu S., Chahal S.M.S. (2019). Incidence of MicroR-4513C/T gene variability in coronary artery disease—A case-control study. Endoc. Metab. Immune Disord. Drug Targ..

[14] Zhou Y., Zhang B., Hao C., Huang X., Li X., Huang Y., Luo Z. (2017). Omentin-A novel adipokine in respiratory diseases. Int. J. Mol. Sci..

[15] Du Y., Ji Q., Cai L., Huang F., Lai Y., Liu Y., Yu J., Han B., Zhu E., Zhang J. (2016). Association between omentin-1 expression in human epicardial adipose tissue and coronary atherosclerosis. Cardiovasc. Diabetol..

[16] Tan Y.L., Zheng X.L., Tang C.K. (2015). The protective functions of omentin in cardiovascular diseases. Clin. Chim. Acta.

[17] Jamshidi J., Ghanbari M., Asnaashari A., Jafari N., Valizadeh G.A. (2017). Omentin Val109Asp polymorphism and risk of coronary artery disease. Asian Cardiovasc. Thorac. Ann..

[18] Nazar S., Zehra S., Azhar A. (2017). Association of single nucleotide missence polymorphism Val109Asp of Omentin-1 gene and coronary artery disease in Pakistani population: Multicenter study. Pak. J. Med. Sci..

[19] Wesener D.A., Wangkanont K., McBride R., Song X., Kraft M.B., Hodges H.L., Zarling L.C., Splain R.A., Smith D.F., Cummings R.D. (2015). Recognition of microbial glycans by human intelectin-1. Nat. Struct. Mol. Biol..

[20] Frostegard J. (2013). Immunity, atherosclerosis and cardiovascular disease. BMC Med..

[21] Rafieian-Kopaei M., Setorki M., Doudi M., Baradaran A., Nasri H. (2014). Atherosclerosis: Process, indicators, risk factors and new hopes. Int. J. Prev. Med..

[22] Stefanadis C., Antoniou C.K., Tsiachris D., Pietri P. (2017). Coronary atherosclerotic vulnerable plaque: Current perspectives. J. Am. Heart Assoc..

[23] Shibata R., Ouchi N., Takahashi R., Terakura Y., Ohashi K., Ikeda N., Higuchi A., Terasaki H., Kihara S., Murohara T. (2012). Omentin as a novel biomarker of metabolic risk factors. Diabetol. Metabol. Syndr..

[24] Shibata R., Ouchi N., Kikuchi R., Takahashi R., Takeshita K., Kataoka Y., Ohashi K., Ikeda N., Kihara S., Murohara T. (2011). Circulating omentin is associated with coronary artery disease in men. Atherosclerosis.

[25] Saely C.H., Leiherer A., Muendlein A., Vonbank A., Rein P., Geiger K., Malin C., Drexel H. (2016). High plasma omentin predicts cardiovascular events independently from the presence and extent of angiographically determined atherosclerosis. Atherosclerosis.

[26] Schaffler A., Zeitoun M., Wobser H., Buechler C., Aslanidis C., Herfarth H. (2007). Frequency and significance of the novel single nucleotide missense polymorphism Val109Asp in the human gene encoding omentin in Caucasian patients with type 2 diabetes mellitus or chronic inflammatory bowel diseases. Cardiovasc. Diabetol..

[27] Greulich S., Chen W.J., Maxhera B., Rijzewijk L.J., van der Meer R.W., Jonker J.T., Mueller H., de Wiza D.H., Floerke R.R., Smiris K. (2013). Cardioprotective properties of omentin-1 in type 2 diabetes: Evidence from clinical and in vitro studies. PLoS ONE.

[28] Yamawaki H., Tsubaki N., Mukohda M., Okada M., Hara Y. (2010). Omentin, a novel adipokine, induces vasodilation in rat isolated blood vessels. Biochemical and Biophysical Research Communications.

[29] Elsaid N.H., Sadik N.A., Ahmed N.R., Fayez S.E., Mohammed N.A.E. (2018). Serum omentin-1 levels in type 2 diabetic obese women in relation to glycemic control, insulin resistance and metabolic parameters. J. Clin. Transl. Endocrinol..

[30] Napoli C., Crudele V., Soricelli A., Al-Omran M., Vitale N., Infante T., Mancini F.P. (2012). Primary prevention of atherosclerosis: A clinical challenge for the reversal of epigenetic mechanisms?. Circulation.

